# Molecular Beacon for Detection miRNA-21 as a Biomarker of Lung Cancer

**DOI:** 10.3390/ijms23063330

**Published:** 2022-03-19

**Authors:** Daniela Alexandre, Bernardo Teixeira, André Rico, Salete Valente, Ana Craveiro, Pedro V. Baptista, Carla Cruz

**Affiliations:** 1 CICS-UBI—Centro de Investigação em Ciências da Saúde, Universidade da Beira Interior, Av. Infante D. 5 Henrique, 6200-506 Covilhã, Portugal; danilex.ct3@gmail.com (D.A.); b.teixeira6200@gmail.com (B.T.); andrerico98@hotmail.com (A.R.); 2 Serviço de Pneumologia do Centro Hospitalar Universitário Cova da Beira (CHUCB), 6200-506 Covilhã, Portugal; msbvalente@gmail.com (S.V.); ana-craveiro@live.com.pt (A.C.); 3 UCIBIO, Department of Life Sciences, Faculdade de Ciências e Tecnologia, Universidade NOVA de Lisboa, 2829-516 Caparica, Portugal; pmvb@fct.unl.pt; 4 i4HB, Associate Laboratory–Institute for Health and Bioeconomy, FCT-NOVA, 2829-516 Caparica, Portugal

**Keywords:** lung cancer, diagnosis, microRNAs, peripheral blood mononuclear cells, molecular beacon

## Abstract

Lung cancer (LC) is the leading cause of cancer-related death worldwide. Although the diagnosis and treatment of non-small cell lung cancer (NSCLC), which accounts for approximately 80% of LC cases, have greatly improved in the past decade, there is still an urgent need to find more sensitive and specific screening methods. Recently, new molecular biomarkers are emerging as potential non-invasive diagnostic agents to screen NSCLC, including multiple microRNAs (miRNAs) that show an unusual expression profile. Moreover, peripheral blood mononuclear cells’ (PBMCs) miRNA profile could be linked with NSCLC and used for diagnosis. We developed a molecular beacon (MB)-based miRNA detection strategy for NSCLC. Following PBMCs isolation and screening of the expression profile of a panel of miRNA by RT-qPCR, we designed a MB targeting of up-regulated miR-21-5p. This MB 21-5p was characterized by FRET-melting, CD, NMR and native PAGE, allowing the optimization of an in-situ approach involving miR-21-5p detection in PBMCs via MB. Data show the developed MB approach potential for miR-21-5p detection in PBMCs from clinical samples towards NSCLC.

## 1. Introduction

In the last three centuries, research efforts provided new approaches for the diagnosis and therapy of cancer [1]. Lung Cancer (LC) has a high impact on cancer incidence and mortality, representing the second most-incident cancer in 2020 [2,3,4]. Numerous factors can contribute to LC development, considering that exposure to multiple specific agents, like mutagens or carcinogens, may stimulate genetic changes [5]. LC staging follows the Classification of Malignant Tumors (TNM) system, where the T stands for tumor, N for node and M for metastasis [6]. Generally, lung cancer is divided into two main types: non-small cell lung cancer (NSCLC), which represents around 75% of cases; and small cell lung cancer (SCLC) with the remaining 25% [7,8]. NSCLC is further distributed in three most common subtypes: adenocarcinoma, the more frequent type, followed by; squamous cell carcinoma; and large cell carcinoma [8]. NSCLC diagnosis is challenging due to ongoing procedure limitations—one reason why a massive research effort is being made, looking for new approaches with a potential application and concomitance with current techniques, for even more accurate NSCLC diagnosis and staging [9,10]. Recent progress shows promising findings in identifying multiple NSCLC-related biomarkers, such as nucleic acids, proteins and circulating tumor cells [11,12,13]. In NSCLC, sundry biomarkers arise as potential diagnostic agents, as well as therapeutic targets [14]. Multiple microRNAs (miRNAs) show an unusual expression profile in NSCLC patients, even in early stages [15,16].

miRNAs are single-stranded non-coding RNAs with about 21–23 nucleotides, which participate in extensive regulatory processes of gene expression via mRNA silencing [17,18]. Besides their intracellular location and related functions, miRNAs can also be found in the extracellular environment, more precisely in biological fluids like plasma and serum [19]. Abnormal miRNA expression is correlated with several diseases, and this dysregulation makes them potential biomarkers for disease diagnostic, subsequent progression and personalized therapeutic strategies. Regarding NSCLC, numerous miRNAs are involved in tumor-related mechanisms, such as uncontrolled cell proliferation, replicative immortality, apoptosis resistance and metastasis, acting either as tumor suppressors or oncogenic miRNAs [20,21,22]. Malignant NSCLC tissue shows dysregulated miRNA profiles in comparison with control counterparts via molecular analysis [21,22]. Plasma, serum and sputum constitute important biological samples for NSCLC-miRNA screening, concerning a less invasive sample collection [23,24,25]. Peripheral blood mononuclear cells (PBMCs) are arising as a potential source of significant biomarkers, including miRNAs [26]. Recent studies are connecting dissimilarities on PBMC signature miRNA profile in several diseases such as cancer and rheumatoid arthritis, among others [26,27,28,29]. Differences in PBMCs miRNA expression under NSCLC condition result from initial immune engagement versus cancer malignancy [30], whose potential for NSCLC diagnosis and therapy is extremely relevant for accurate tumor identification and personalized medicine approaches. From correct diagnostic to prognosis prediction, miRNA-specific profile screening plays a critical role in NSCLC, as biological markers capable of differentiating NSCLC subtypes, discriminating between primary and metastatic tumors, arise as a basis of emerging minimally invasive methods [21,30].

MB technology arises as a potential bioanalytical system in the biotechnology field, presenting a wide group of useful features and advantages. One of the most interesting features of MBs relies on the capacity to detect nucleic acids. The MB stem-loop characteristic structure brings the fluorophore and the quencher together via stem sequence complementarity, resulting in fluorescence quenching due to both the fluorophore and quencher proximity. Target complementary sequences (DNA or RNA) interact with the MB loop region, promoting a strong intermolecular connection and stem split. Upon hybridization, stem sequences separate alongside with the fluorophore and the quencher at the end of each strand, which will guide to fluorescence recovery signal [31,32]. Biosensors with surface-immobilized MBs trend in molecule screening. For example, a molecular aptamer beacon (MAB) with G-quadruplex (G4) formation capable of integrating a micro-chip surface showed ability to detect the prostate cancer protein nucleolin [31,33]. Recently, a MB assay was developed for severe acute respiratory syndrome coronavirus 2 detection [34].

miRNAs detection based on MB fluorescence approaches can represent a powerful tool toward groundbreaking achievements in NSCLC diagnosis. In comparison with the RT-qPCR mainstay procedure for miRNA detection in biological samples, MB approaches are efficient tools for miRNA monitoring by being a more cost-effective option, a faster and handier assay due to a quick in-situ MB hybridization within a specific target, and representing a more affordable alternative regarding all the devices and reagents needed in RT-qPCR [34,35,36]. MB development targeting miR-21 and miR-155 is reported, considering that they play a critical role in multiple cancers, including NSCLC [37,38,39,40,41,42]

In this work, NSCLC-miRNAs were profiled in PBMCs and selected for detection in clinical samples. A MB-based approach targeting miR-21-5p was developed for NSCLC diagnosis, which generates a proportional fluorescent signal to miR-21-5p concentration.

## 2. Results

### 2.1. miRNA Profiling

A panel of NSCLC-related miRNAs was selected for expression analysis in PBMCs from NSCLC and healthy individuals via RT-qPCR, using miRNAs 103a-3p and 191-5p as control genes. NSCLC-related miRNAs 21-3p, 21-5p, 92b-5p, 150-3p, 155-3p, 155-5p, 181a-5p and 3662 were profiled in samples retrieved from NSCLC and healthy individuals. Additional miR-21-5p expression analysis was performed in MRC-5 normal lung and A549 NSCLC cell lines and normalized to U6 housekeeping gene. Concerning PBMC miRNA expression analysis, a preliminary miRNA screening was conducted, comprising 12 healthy control individuals and 23 NSCLC patients for all miRNAs. Tested samples were increased up to 25 healthy controls and 50 NSCLC patients for miRNAs 21-5p, 92b-5p, 150-3p and 155-3p.

**Figure 1:**
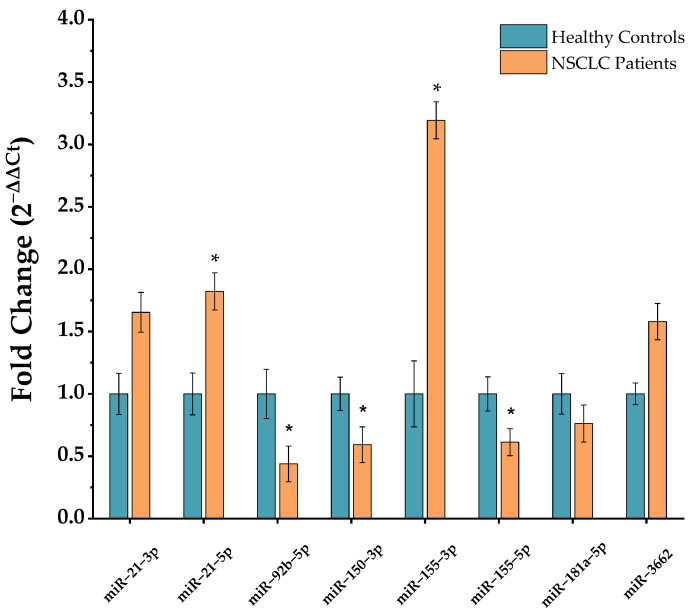
Selected-miRNA relative expression in NSCLC and healthy control PBMCs. Data were normalized to housekeeping miRNAs 103a-3p and 191-5p, and further NSCLC miRNA expression calculated relatively to a healthy control expression value of 1 (mean ± SD, * *p* < 0.05). Data population after outlier exclusion for multiple miRNAs was: miR-21-3p (control *n* = 19, NSCLC *n* = 23); miR-21-5p (control *n* = 21, NSCLC *n* = 50); miR-92b-5p (control *n* = 20, NSCLC *n* = 45); miR-150-3p (control *n* = 19, NSCLC *n* = 45); miR-155-3p (control *n* = 20; NSCLC *n* = 45); miR-155-5p (control *n* = 12; NSCLC *n* = 21); miR-181a-5p (control *n* = 10, NSCLC *n* = 18); and miR-3662 (control *n* = 10; NSCLC *n* = 18).

Figure 1 exhibits RT-qPCR normalized miRNA profiling results in NSCLC and control PBMCs. miRNAs 21-3p, 21-5p, 155-3p and 3662 showed an up-regulated profile in NSCLC, wherein miR-21-3p indicated a 1.65-fold change, miR-21-5p a 1.82-fold change, miR-155-3p a 3.19-fold change and 3662 a 1.59-fold change over healthy regular expression. In contrast, miRNAs 92b-5p, 150-3p, 155-5p and 181a-5p revealed a lower expression in NSCLC relative to a healthy control population, with a 0.44-fold change for miR-92b-5p, a 0.59-fold change for 150-3p, a 0.61-fold change for 155-5p and a 0.76-fold change for 181a-5p. Furthermore, miRNAs 21-5p, 92b-5p, 150-3p, 155-3p and 155-5p showed significant differences between NSCLC and healthy control populations.

As such, miR-21-5p, which is also over-expressed in the model A549 NSCLC cell line with a 2.03-fold change compared to MRC-5 normal lung cell line (Figure S1), was selected for MB design, as well as further in-situ miRNA detection approach development using these cells lines as models.

### 2.2. MB Design and Biophysical Characterization

**Figure 2:**
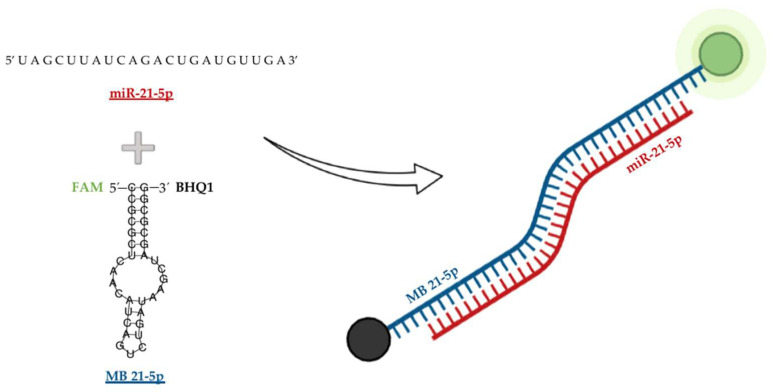
Designed MB 21-5p targeting miR-21-5p folding structure simulation with the respective fluorophore FAM (red) and quencher BHQ1 (black).

Hybridization kinetics and dynamics were studied for the designed MB 21-5p targeting miR-21-5p. MB 21-5p design comprised a stem region coupled with a donor-quencher pair (5′-FAM and BHQ1-3′) and a loop region corresponding to the miR-21-5p reverse complementary sequence. Upon structure software simulations, MB 21-5p folding configuration displayed both the stem and the loop region, with a shorter stem at the top of the loop region, resulting from base complementarity inner bonding on the miR-21-5p specific complementary sequence (Figure 2).

**Figure 3:**
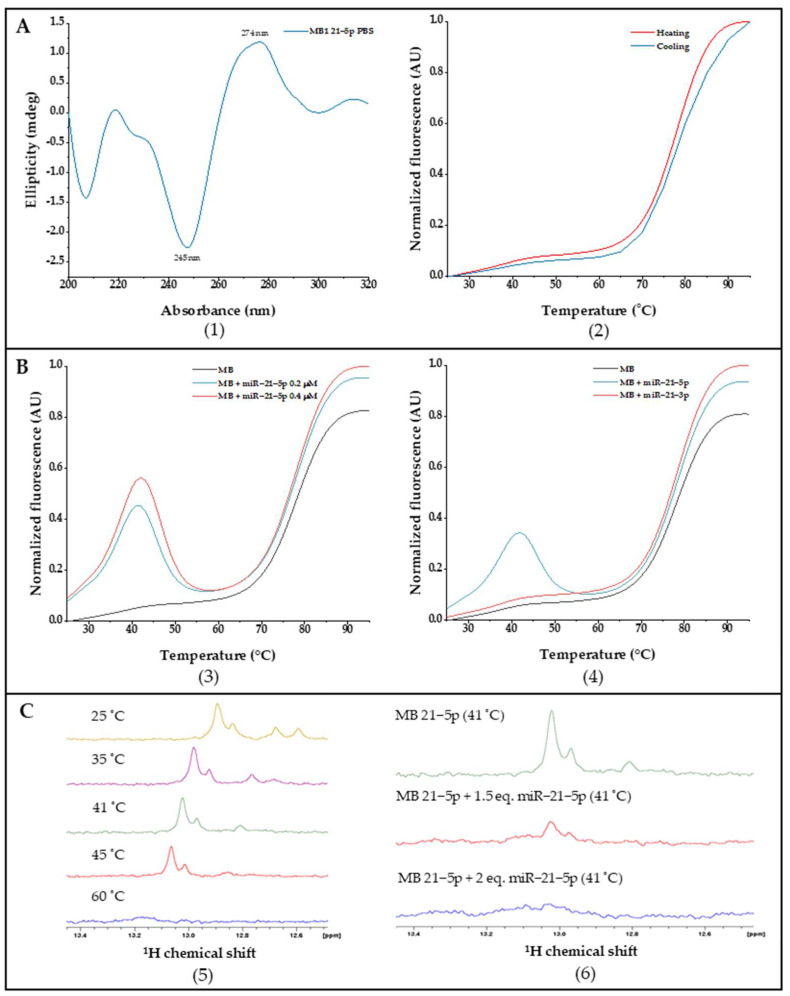
(**A**). (**1**) CD spectra of MB 21-5p in PBS; (**2**) MB 21-5p FRET and reverse FRET melting curves normalized to [0, 1]. (**B**). MB 21-5p FRET and reverse FRET melting curves with (**3**) specific miR-21-5p and (**4**) non-specific miR-21-3p normalized to [0, 1]. (**C**). ^1^H NMR spectra of (**5**) MB 21-5p with temperature variation and (**6**) MB 21-5p with miR-21-5p at the optimal hybridization temperature.

MB 21-5p structure characterization was studied by CD spectroscopy. Figure 3A(1) showed a signature of double helix secondary structure profile with a negative band at approximately 245 nm, as well as a positive band at 274 nm.

Folding and conformational dynamics from MB 21-5p were assessed by FRET melting experiments, where fluorescence is monitored over temperature variation, enabling MB configuration track from closed stem-loop until random coil via donor-quencher division through a temperature increase and a further donor fluorescent signal. Throughout FRET melting assays we obtained a *T_m_* of 77 °C for MB 21-5p, which also exhibited a similar FRET and reverse FRET curve profile (Figure 3A(2)). Moreover, interaction studies between MB 21-5p and the target 21-5p and the non-specific miR-21-3p were also carried out through FRET melting assays. MB 21-5p (0.2 μM) with the specific miR-21-5p synthetic sequence (0.2 μM and 0.4 μM) exhibited a fluorescence peak at 41 °C, which is slightly stronger for miR-21-5p higher concentrations. At 41 °C, MB 21-5p with miR-21-5p reaches a higher fluorescence value, which represents the maximum hybridization MB-miRNA rate (Figure 3B(3)). As observed in Figure 3B(4), by adding a non-specific miRNA sequence (miR-21-3p) to the MB 21-5p, the FRET melting curve profile behavior was similar to the single MB 21-5p.

Next, NMR spectroscopy was performed to check the hybridization of MB 21-5p with miR-21-5p target. MB 21-5p ^1^H NMR spectra revealed specific Watson-Crick GC base pairing signals at 13 ppm related with the MB 21-5p stem region formed in the MB signature hairpin folded configuration. A temperature increase led to a signal reduction and further vanishing at 60 °C, characteristic of the stem region breakdown and MB unfold (Figure 3C(5)). In Figure 3C(6), the imino signal disappearance is visible with the addition of the specific miR-21-5p target to MB 21-5p.

Native PAGE was performed to confirm MB 21-5p hybridization and specificity, in which MB with the synthetic miR-21-5p produced a higher weight product corresponding to the complex formation between MB and miR. Furthermore, native PAGE results for MB 21-5p with the non-specific miR-21-3p did not generate a higher weight complex, confirming the MB specificity for the correspondent target. (Figure S2). MB hybridization and specificity was further confirmed by fluorescence for concept validation before biological sample testing (Figure S3).

### 2.3. Using the MB for Specific miRNA-21-5p Detection in Clinical Samples

The developed MB 21-5p performance for detection of miRNA-21-5p was firstly assessed using total RNA extracted from cancer and normal model cell lines. MB fluorescence for A549 cell line, which overexpresses miRNA-21-5p, showed higher fluorescence intensity when compared to MRC-5 cell line (Figure S4).

**Figure 4:**
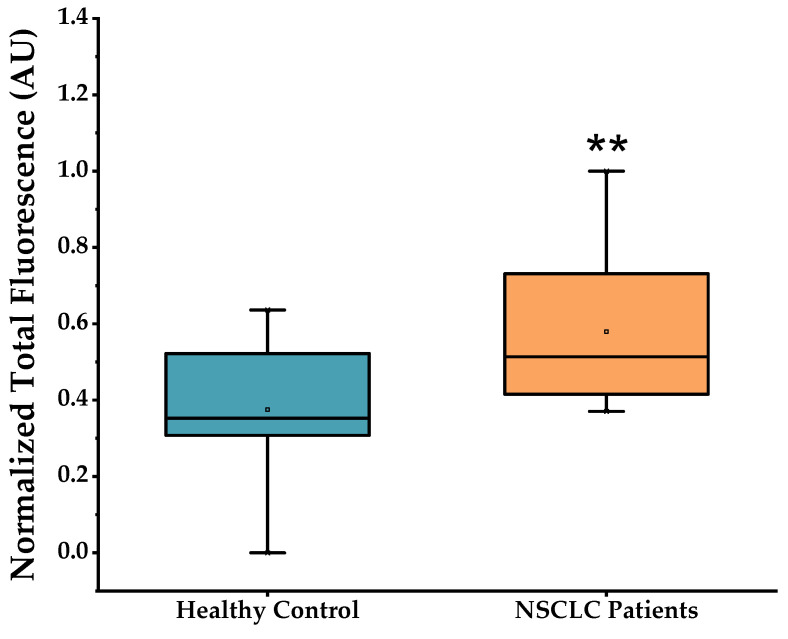
MB 21-5p hybridization assays with RNA from healthy control and NSCLC PBMCs (mean ± SD, ** *p* < 0.01). Data corrected for basal fluorescence and normalized to [0, 1]. Samples in study comprised: healthy control group (*n* = 15); NSCLC group (*n* = 24).

Figure 4 represents the fluorescence signal arising from MB 21-5p in PBMCs from patients and control clinical samples. A significant higher fluorescence is visible in NSCLC PBMC samples compared to controls. This is in total agreement with the overexpression profile of miR-21-5p over-expressed profile in NSCLC characterized by RT-qPCR (see above). MB 21-5p miR-21-5p detection robustness was measured via ROC curve analysis, in which the developed method was able to discriminate between NSCLC patients with 100% sensitivity (true positives) and 55.3% specificity (true negatives) (Figure S5). By excluding NSCLC stage IV patients, the MB 21-5p developed approach exhibited a higher fluorescence proclivity for NSCLC PBMCs in stages I, II and III, compared to healthy volunteers, while still being capable of identifying true positive cases with 100% sensitivity and true negative cases with 55.3% specificity (Figures S6 and S7).

Additionally, the miR-21-5p expression can be detected in the six patients presenting TNM stages I and II with the same sensitivity and true negative specificity determined previously (Figure S7).

## 3. Discussion

NSCLC is generally diagnosed in advanced stages, so therefore the demand for novel straightforward methods for diagnosis and staging is crucial [9,10]. Regarding NSCLC, numerous miRNAs are involved in tumor-related mechanisms by acting either as tumor suppressor or oncogenic miRNAs [21,22]. miRNA expression profile in PBMCs arises as a potential biomarker source in NSCLC [30]. We developed an in situ methodology based on the principle of MB recognition of specific target miRNA associated with NSCLC through hybridization and subsequent fluorescence emission.

A panel of NSCLC-related miRNAs, comprising miRNAs 21-3p, 21-5p, 92b-5p, 150-3p, 155-3p, 155-5p, 181a-5p and 3662, were screened in PBMCs from NSCLC and healthy individuals. The miR-21-3p (1.65-fold change) and miR-21-5p (1.82-fold change) are both up-regulated in PBMCs of NSCLC patients, corroborating with miR-21 expression determined in plasma [39,40,41,42,43]. Our results correlate well with those attained in previous studies showing over-expression of miR-21 in NSCLC PBMCs as a possible biomarker [25,44].

Obtained results on the remaining miRNAs (miR-92b-5p, miR-150-3p, miR-155-3p, miR-155-5p, miR-181a-5p and miR-3662) show an up- or down-regulated miRNA expression profile in NSCLC PBMCs. The selected miRNAs have their dysregulation reported in NSCLC tissue, plasma and serum [45,46,47,48,49]. According to Ma et al., however, there is no overlap between the miRNA expression profile in PBMCs with the profile in NSCLC tissue and extracellular fluids [30]. Besides obtained results on the miRNA expression study in PBMCs through RT-qPCR, the cohort of NSCLC patients and healthy control population is limited. Upon the cohort characterization analysis, differences are visible in average age and smoking status between both populations, as well as for NSCLC stage in the NSCLC group. The NSCLC population is considerably older and have a higher percentage of smokers, and TNM stages III and IV represent most total NSCLC cases.

For the MB studies, miR-21-5p was selected due to the significant up-related relative expression (1.82-fold change) in NSCLC PBMCs and to the absolute expression, showing higher concentrations compared to miR-155-3p (miR-21-5p Ct amplification mean = 15.85; miR-155-3p Ct amplification mean = 31.87). The A549 NSCLC cell line also exhibited an over-expression of miR-21-5p (3.33-fold change) relative to MRC-5 normal lung cell line [43,50]. 

MB 21-5p was designed to target miR-21-5p through a miR-21-5p specific reverse complementary loop region and a stem region labelled with the fluorophore FAM and BHQ1 quencher. MB 21-5p biophysical characterization by CD spectroscopy showed that the MB 21-5p CD spectra resemble the MB stem region in the double helix conformation [51,52]. *T_m_* of MB 21-5p determined by FRET melting in PBS was 77 °C and the MB 21-5p reverse FRET melting curve was similar to the FRET melting curve. FRET melting results on MB 21-5p with miR-21-5p showed that MB 21-5p displayed a signature 41 °C fluorescence peak, which represents the temperature for maximum hybridization efficiency. When along with miR-21-3p, MB 21-5p depicted the same FRET curve profile as the MB 21-5p, suggesting no hybridization with miR-21-3p. Furthermore, this assay provided evidence that supports MB 21-5p specificity for miR-21-5p.

^1^H NMR spectra of MB depicted two signals between 12.6–12.8 ppm and 13 ppm that correspond to the hairpin region and stem of the MB, respectively. When temperature increases, it appears that the first signals to vanish are the 12.6–12.8 ppm loop region, indicating an unfold of the loop towards the stem. Signature MB signals are no longer visible at 60 °C, suggesting the total MB 21-5p unfold into a random coil [53,54,55,56,57]. The addition of miR-21-5p to MB 21-5p led to the signal disappearance of the stem-loop, suggesting hybridization of MB 21-5p with miR-21-5p.

The MB 21-5p hybridization was also studied by native PAGE at a hybridization temperature of 41 °C, showing specific and robust MB hybridization to the target. Furthermore, there was no indication of hybridization of MB 21-5p to miR-21-3p, acting as non-related sequence target. Moreover, the fluorescence assay with MB 21-5p with synthetic miR-21-5p and miR-21-3p further supports specificity of recognition.

MB 21-5p was capable of specifically detecting miR-21-5p in the model cancer cell line (A549) over normal lung cell line. Significant higher fluorescence signals were also detected in PBMCs of NSCLC patients using our MB-based approach, in line with data attained by RT-qPCR.

We then used the optimized MB to screen the clinical samples of NSCLC and healthy individuals. The developed in situ MB-based approach for miR-21-5p detection produced noteworthy results in biological samples, more specifically in total RNA from PBMCs, which make it a valuable tool for NSCLC molecular diagnostics, presenting a 100% sensitivity capacity and a 55.3% specificity. The approach remained as sensitive (100%) and specific (55.3%) for NSCLC stages I, II and III, showing a higher fluorescence tendency in cases of NSCLC. However, care should be taken when assessing these data, since some false positives could be detected, probably due to the high sensitivity of the MB and/or leaky fluorescence signals under real sample conditions.

## 4. Materials and Methods

### 4.1. Patients and Samples

Fresh human blood samples were collected from 50 patients previously diagnosed with LC and 25 healthy volunteers with no current or previous malignant disease, according to the protocol approved by the Ethics Committee of University Hospital Center Cova da Beira (ref. 35/2019). All tested subjects gave written informed consent. The LC patients include different histological types at different clinical stages (Table 1).

Fresh whole blood samples were collected in ethylenediaminetetraacetic acid (EDTA)-coated tubes (Sigma-Aldrich, Missouri, SL, USA). The isolation of PBMCs was performed as follows. Firstly, the blood samples were briefly centrifuged in Beckman Coulter Allegra X-22R, SX4250 rotor at 3000 rpm (Beckman Coulter, Brea, CA, USA) for 15 min, at room temperature. After centrifugation, the plasma fraction was removed and an equal volume of 1× phosphate-buffered saline (PBS) was added to the tube, followed by gentle mixing by inverting the tube several times. The mixture was then gently transferred into a 50 mL polypropylene tube containing an equal volume of Pancoll (PAN-Biotech, Aidenbach, Germany) separating solution, and centrifuged at 2200 rpm for 30 min at room temperature. The PBMCs (ring) formed in the interphase between Pancoll and blood fraction was aspirated using a plastic Pasteur pipette, and transferred to a new 50 mL polypropylene tube. Then, 1× PBS was added to the 50 mL polypropylene tube and the mixture was centrifuged at 1800 rpm for 10 min at 4 °C. After discarding the supernatant, the pellet was resuspended in 10 mL of 1× PBS, and centrifuged again at 1500 rpm for 10 min at 4 °C. The supernatant was discarded, the pellet was resuspended in 10 mL of pre-warmed RBC lysis solution and gently stirred at 37 °C for 10 min. The sample was then centrifuged at 1500 rpm for 10 min at room temperature. The resulting pellet was then washed two times with 1× PBS by centrifugation at 1500 rpm for 10 min at room temperature. Finally, the PBMCs were resuspended in 1 mL of 1× PBS for cell count and stored at −80 °C until further use.

To compare the results obtained from PBMC of LC patients, the human lung adenocarcinoma cell line A549 and normal human lung fibroblasts (MRC-5) cell line were used as the cytological model. All cells were purchased from the American Type Culture Collection (ATCC, Manassas, VA, USA) cell bank. The cells were cultured in appropriate medium, supplemented with 10% (*v*/*v*) fetal bovine serum (FBS) and 1% (*v*/*v*) penicillin-streptomycin. Cultures were grown and maintained in a humidified atmosphere at 37 °C in and 5% CO_2_, and collected and stored at −80 °C until further use.

### 4.2. RNA Isolation and Quantitative RT-PCR

To analyze the different miRNAs expression of samples, RT-qPCR analysis was performed.

Small RNA molecules with size <200 nucleotides were extracted and purified from all samples using the miRNeasy Mini Kit (Qiagen, Hilden, Germany), according to the kit instructions. Upon 30 µL RNA elution, the samples were immediately placed on ice and the RNA concentration was measured in the Nano Photometer (IMPLEN, Munich, Germany). A 260/280 ratio represents protein contamination, wherein values superior to 1.7 are good and relate with no protein contamination.

First Strand cDNA synthesis was performed using miRCURY LNA RT Kit (Qiagen, Hilden, Germany), and 200 ng total RNA of each sample was reverse transcribed according to the kit instructions. The reaction included 1 μL of 10× RT enzyme mix, 2 μL of 5× RT reaction buffer, and a corresponding volume for 200 ng RNA and RNase-free water to make a total volume of 10 μL. The RT reaction was incubated for 60 min at 42 °C, followed by 5 min at 95 °C, and finally stopped at 4 °C for storage.

For quantitative analysis of gene expression, RT-qPCR amplification of cDNA was performed using miRCURY LNA SYBR^®^ Green Kit (Qiagen, Hilden, Germany) on a CFX Connect™ Real-Time PCR Detection System (Bio-Rad, Hercules, CA, USA). The miRCURY LNA miRNA PCR assay primers used are described in Table 2. Cycling conditions for RT-qPCR involved an initial reaction heat activation at 95 °C for 2 min, followed by 2-step cycling comprising a denaturation during 10 s at 95 °C and a further annealing/extension for 60 s at 56 °C. Previous steps were repeated for 40 cycles.

Each sample was run in duplicate from three different experiments (in the case of cell lines), and the relative quantification of gene expression was based on the comparative threshold cycle (CT) method, in which the quantity of transcripts is determined as 2−(ΔCT target−ΔCT control), normalized to levels of U6 housekeeping results, and are expressed relative to the healthy samples (means ± SEM).

### 4.3. MB Design and Synthetic Sequence Preparation

The MB was designed for the targeting of miR-21-5p with the RNAFold prediction software [58]. The miR-21-5p synthetic sequence (5′-UAGCUUAUCAGACUGAUGU UGA-3′) and MB 21-5p were obtained from Eurofins (Eurofins Genomics, Louisville, KY, USA) with HPLC-grade purification. As explained above, MB miRNA-based detection approaches use the FRET phenomenon to yield a fluorescent signal upon MB hybridization with specific miRNA, due to donor and acceptor separation.

To the equivalent miR-21-5p DNA sequence were added each 5′ and 3′ ends six nucleotide CG rich sequences, in order to form a stable duplex stem that closes the loop region and subsequently forms the MB characteristic configuration. The MB was purchased labelled with fluorescein (FAM) and quencher (BHQ1) at the 5′ and 3′ ends, respectively.

Stock solutions of MB and miRNA synthetic sequences were prepared using nuclease-free water and stored at −20 °C until further experiments. The concentration was determined by a Thermo Scientific™ Evolution 220 UV-vis spectrophotometer (Thermo Fisher Scientific, Waltham, MA, USA) at 260 nm using the molar extinction coefficients (ε) provided by the manufacturer.

### 4.4. CD Spectroscopy

CD spectra were acquired in a Jasco J-815 spectrometer (JASCO Corporation, Tokyo, Japan) using a Peltier temperature controller (model CDF-426S/15). The MB 21-5p solution, at a concentration of 10 μM, was dissolved in 10 mM of lithium cacodylate (Sigma-Aldrich, Missouri, SL, USA) and annealed as already described. CD spectra acquisitions were performed in a 1 mm path-length quartz cuvettes at 20 °C, with a 200–340 nm spectral scan range, scan speed of 200 nm/min, 1 nm bandwidth and 1s integration time over 4 accumulations. CD melting experiments were also performed, and the MB denaturation study was analyzed from across different temperatures, ranging from 20 to 100 °C with a heating rate of 2 °C/min, while monitoring the wavelength of maximum ellipticity at 274 nm. Data were converted into folded fraction (θ) plots fitted to a Boltzmann distribution using OriginPro2018 (OriginLab, Northampton, MA, USA).

The melting temperature (*T*_m_) was determined from a two-state transition model where CD is the ellipticity at 274 nm at each temperature, and CDmin and CDmax are the lowest and highest ellipticities, respectively.

### 4.5. Fluorescence Resonance Energy Transfer (FRET) Melting

FRET-melting and reverse FRET-melting experiments were performed for MB structure and dynamics study.

The experiments were performed in a 96-well plate using a CFX Connect™ Real-Time PCR Detection System (Bio-Rad, Hercules, CA, USA), equipped with a FAM filter (λ_ex_ = 495 nm; λ_em_ = 520 nm). The MB 21-5p was prepared in 10 mM lithium cacodylate (pH 7.2) at a concentration of 0.2 μM, and the MB solution was firstly annealed at 95 °C for 3 min and then cooled down at room temperature. For interaction studies, their corresponding miR-21-5p synthetic sequence was added to the wells, as well as the mismatched miR-21-3p, and carried out by FRET and reverse FRET-melting assays. Solutions containing MB and the miRNA were prepared in a broad range of different concentrations and MB/miRNA ratios. The thermocycler was set to perform a stepwise increase of 1 °C every 1 min, from 25 °C to 95 °C, and the FAM emission was measured after each step. The samples were heated up to 95 °C and further cooled down to 25 °C in the following reverse FRET-melting step. Fluorescence output data were normalized and fitted to Boltzmann distribution using OriginPro2018 (OriginLab, Northampton, MA, USA). MB Tm was calculated based on the corresponding temperature value to half of the total fluorescence reached.

### 4.6. Nuclear Magnetic Resonance Spectroscopy

Standard 1H NMR spectra were recorded on a Bruker Avance III 600 MHz spectrometer (Bruker, Billerica, MA, USA) equipped with a 37 QCI CryoProbe at 25 °C. The NMR zgesgp pulse sequence was used to suppress the water signal. A 200 μL of MB 21-5p solutions were prepared in Mili-Q water, comprising 50 μM of MB and 10 mM lithium cacodylate, and supplemented with 10% of deuterium water (D_2_O). Previous solutions were set up in a 3 mm NMR tubes and annealed as described above. MB 21-5p NMR spectra were acquired at 25 °C, 40 °C and 60 °C for the MB alone and MB with miR-21-5p synthetic sequence. After this, NMR titration with Mg^2+^ was performed by adding increasing amounts of MgCl_2_ to the 3 mm tube, corresponding to concentrations of 50, 100, 200, 300 and 500 μM. All spectra were acquired and processed with the software Topspin 3.1 (Bruker, Billerica, MA, USA). Images were prepared using MestReNova (Mestrelab, Santiago de Compostela, Spain). Chemical shifts (δ) were measured in ppm.

### 4.7. Non-Denaturing Polyacrylamide Gel Electrophoresis

Electrophoresis procedure was used to study MB-miRNA hybridization through molecular mass separation.

Non-denaturing polyacrylamide gel (15%) was prepared and two sets of samples were prepared, involving in each one 5 μM of miRNA, 5 μM of MB 21-5p in study, 5 μM of MB with one equivalent of miRNA (5 μM) and 5 μM of MB with two equivalents of miRNA (10 μM). To the four solutions were added 15% of sucrose. Thereafter, each set of solutions was incubated at 41 °C for 10 min, to allow hybridization between MB and miRNA. Upon incubation, 20 μL of each sample were loaded into the gel according to a designed template, followed by the electrophoresis running step at 130 V for 1 h at room temperature. After electrophoresis, the gels are placed in fixing solution for 1 h and then emerged in a staining solution for 20–40 min, under gentle agitation and with samples visualized.

### 4.8. Fluorescence Assays—miRNA Detection

The fluorescence studies were performed on a FluoroMax 4 fluorometer (HORIBA, Kyoto, Japan) equipped with a temperature control system. 1 μM of MB 21-5p was annealed as previously described, and then loaded into a high-precision quartz suprasil cuvette (light path 10 mm × 4 mm; Hellma Analytics, Müllheim, Germany). Spectra measurement settings were applied under fluorophore FAM characteristics (λ_ex_ = 535 nm, λ_em_ = 520 nm), and spectra was acquired in a 25–95 °C temperature range and vice versa, with 3 average scans. For these experiments, 1 μM of miR-21-5p synthetic sequence was added, and the same procedure was performed. MBs optimal hybridization conditions were screened before biological assays. MB and MB + miRNA solutions with different MB concentrations were prepared in a 0.2 mL PCR tube on ice, using 1× PBS. Samples were placed in a T100 Thermal Cycler (Bio-Rad, Hercules, CA, USA), where they passed through an initial annealing step at 95 °C for 10 min and a hybridization step involving different temperature and time ranges. Samples were then pipetted into a 96-well microplate for fluorescence-based assay and carefully homogenized for 3 min, and fluorescence was measured in a SpectraMax Gemini XPS Microplate Reader (Molecular Devices, San José, CA, USA). Templates were designed regarding the different conditions in study, and for each condition, it was set a MB and a MB + miRNA well. A control sample with only MB is included. Biological assays were performed under optimized conditions from screening experiments. MB was tested with total RNA from cell lines and PBMCs. Solutions concerning MB and biological samples were prepared on ice, and then loaded into a 96-well microplate. The plate was homogenized for 3 min, and fluorescence was measured at different temperatures up to 45 °C, until reaching a fluorescence peak.

### 4.9. Statistic Analysis

Data from assays comprising more than one sample were presented as mean ± standard deviation (SD). Statistics on quantitative RT-qPCR data and final fluorescence results for MB-miRNA detection via MB 21-5p targeting miR-21-5p with total RNA were analyzed in OriginPro2018 (OriginLab, Northampton, MA, USA) by Two sample t Test with standard deviation (SD), where significant differences with a 95% (*p* < 0.05), 99% (*p* < 0.01 and 99.9% (*p* < 0.001) confidence interval were also studied. Additionally, developed MB-based strength was examined in the medical statistics software MedCalc (MedCalc, Ostend, Belgium) via receiver operating characteristic (ROC) curve analysis. This data evaluation allows the ability of the approach to identify true NSCLC positive cases (sensitivity), true negatives (specificity) and discrimination robustness via the area under the curve (AUC) value.

## 5. Conclusions

Our miRNA profiling data indicated an up-regulation of miRNAs 21-3p, 21-5p, 155-3p and 3662 in NSCLC PBMC samples, although miRNAs 92b-5p, 150-3p, 155-3p and 181a-5p exhibited an under-expressed profile. miR-21-5p was selected as MB-target due to RT-qPCR result analysis regarding absolute and relative quantification, where miR-21-5p was up-regulated in PBMCs (1.82-fold change), and in higher concentrations compared to other potential targets.

The designed MB 21-5p demonstrated an ability to hybridize towards the miR-21-5p specific target by different techniques. Furthermore, MB 21-5p also exhibited hybridization specificity to miR-21-5p over miR-21-3p. This MB 21-5p showed the capability to detect the specific target with 100% sensitivity and 55.3% specificity, even in NSCLC stages I, II and III.

Although these results constitute prominent advances towards miR-21-5p in NSCLC, further experimental assays comprising more patients and additional procedure optimizations are needed. Future work perspectives include a larger NSCLC and healthy cohort to validate results and the final MB-based approach. Future cohort efforts involve an older healthy population with smoking history and more NSCLC early-stage cases (TNM I and II). Still, the developed MB-based methodology presents fast, less expensive and easy to assess results in NSCLC clinical samples than RT-qPCR, and a multiple miRNA detection array could constitute a valuable approach for a more specific NSCLC diagnosis.

## Figures and Tables

**Table 1:** Characterization of lung cancer patients and healthy individuals enrolled in the study.

	NSCLC (N = 50)	HEALTHY INDIVIDUALS (N = 22)
**AGE (YEARS)**		
Mean ± SD	69.92 ± 8.08	29.5 ± 8.07
**GENDER**		
Male	37 (74%)	9 (41%)
Female	13 (26%)	13 (59%)
**SMOKING STATUS**		
Nonsmoker	20 (40%)	19 (86%)
Smoker	16 (32%)	3 (14%)
Ex-smoker	14 (28%)	0 (0%)
**HISTOLOGICAL TYPE**		
Adenocarcinoma	36 (72%)	NA
Squamous cell carcinoma	10 (20%)	NA
Large cell carcinoma	4 (8%)	NA
**TNM STAGE**		
I	2 (4%)	NA
II	4 (8%)	NA
III	8 (16%)	NA
IV	36 (72%)	NA
**METASTASIS**		
Yes	33 (66%)	NA
No	17 (34%)	NA

**Table 2:** miRCURY LNA miRNA PCR assay primers for RT-qPCR miRNA screening.

Name (miRCURY Primers)	Role	miRNA Target Sequence (from 5′ to 3′)
hsa-miR-103a-3p	Housekeeping	AGCAGCAUUGUACAGGGCUAUGA
hsa-miR-191-5p	Housekeeping	CAACGGAAUCCCAAAAGCAGCUG
U6snRNA hsa-miR-21-3p	Housekeeping NSCLC-related	CACGAATTTGCGTGTCATCCTT CAACACCAGUCGAUGGGCUGU
hsa-miR-21-5p	NSCLC-related	UAGCUUAUCAGACUGAUGUUGA
hsa-miR-92b-5p	NSCLC-related	AGGGACGGGACGCGGUGCAGUG
hsa-miR-150-3p	NSCLC-related	CUGGUACAGGCCUGGGGGACAG
hsa-miR-155-3p	NSCLC-related	CUCCUACAUAUUAGCAUUAACA
hsa-miR-155-5p	NSCLC-related	UUAAUGCUAAUCGUGAUAGGGGUU
hsa-miR-181a-5p	NSCLC-related	AACAUUCAACGCUGUCGGUGAGU
hsa-miR-3662	NSCLC-related	GAAAAUGAUGAGUAGUGACUGAUG

## Data Availability

Not applicable.

## Supplementary Materials

The following supporting information can be downloaded at: https://www.mdpi.com/article/10.3390/ijms23063330/s1.
