# Supplementary material for: Molecular Beacon for Detection miRNA-21 as a Biomarker of Lung Cancer
Source: Int J Mol Sci. 2022 Mar 19;23(6):3330. doi: 10.3390/ijms23063330 (PMC8955680; doi:10.3390/ijms23063330)
Supplement: Supplementary file 1 [file ijms-23-03330-s001.zip › ijms-1619983-supplementary.pdf]

Supporting Information

Molecular Beacon for detection miRNA-21 as a biomarker of lung cancer

Daniela Alexandre[1,†], Bernardo Teixeira[1,†], André Rico[1], Salete Valente[2], Ana Craveiro[2], Pedro V. Baptista[3,4] and Carla Cruz [1,*]

[1]CICS-UBI - Centro de Investigação em Ciências da Saúde, Universidade da Beira Interior, Av. Infante D. 5 Henrique, Covilhã, Portugal;
[2]Serviço de Pneumologia do Centro Hospitalar Universitário Cova da Beira (CHUCB), Covilhã, Portugal;
[3]UCIBIO, Department of Life Sciences, Faculdade de Ciências e Tecnologia, Universidade NOVA de Lisboa, 2829-516 Caparica, Portugal.
[4]i4HB, Associate Laboratory - Institute for Health and Bioeconomy, FCT-NOVA

[*]Correspondence: Corresponding author email: carlacruz@fcsaude.ubi.pt
[†]These authors contributed equally to this work.

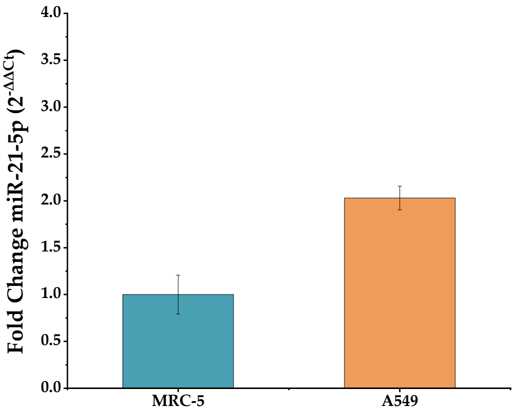

Figure S1. miRNA 21-5p relative expression in MRC-5 normal lung and A549 NSCLC cell lines. A549 miRNA expression was performed through RT-qPCR assays in duplicate, normalized, and standardized to MRC-5 control expression value 1 (mean ± SD, n=5).

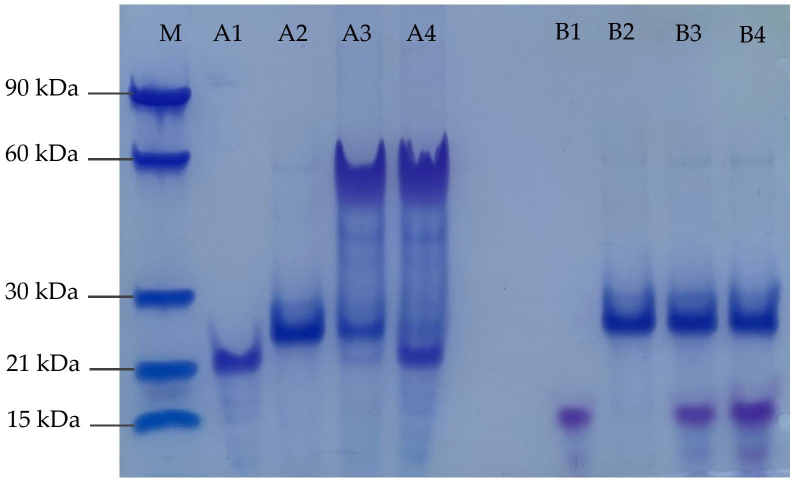

Figure S2. MB 21-5p hybridization native PAGE gel results with previous 41 °C incubation. M: Weight marker; A: MB 21-5p + specific miR-21-5p set; B: MB 21-5p + non-specific miR-21-3p set; 1: miRNA (5 µM); 2: MB 21-5p (µM); 3: MB 21-5p (5 µM) + miRNA (5µM); 4: MB 21-5p (5 µM) + miRNA (10 µM).

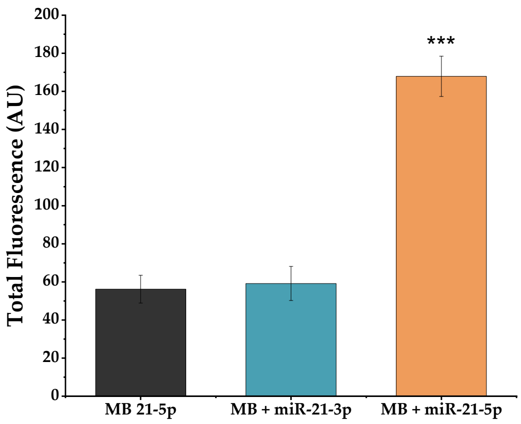

Figure S3. MB 21-5p hybridization with miR-21-5p and non-specific miR-21-3p (mean ± SD, ****p* < 0.001). Fluorescence is represented in total values measured in the plate reader (n=3).

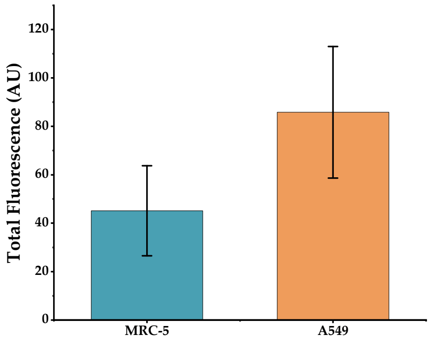

Figure S4. MB 21-5p MRC-5 and A549 RNA hybridization assays (mean ± SD). Fluorescence data is represented with MB 21-5p basal fluorescence discounted (n=3).

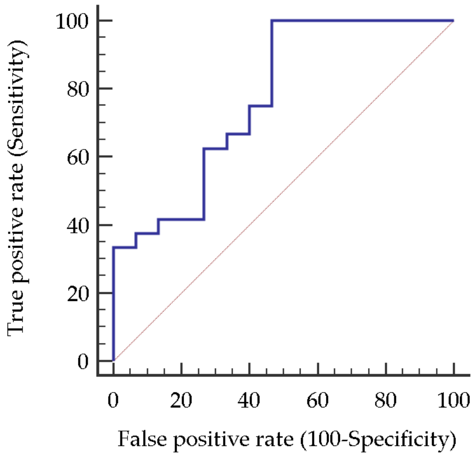

Figure S5. ROC curve analysis on the developed MB 21-5p hybridization approach for miR-21-5p detection in RNA from healthy control and NSCLC PBMCs.

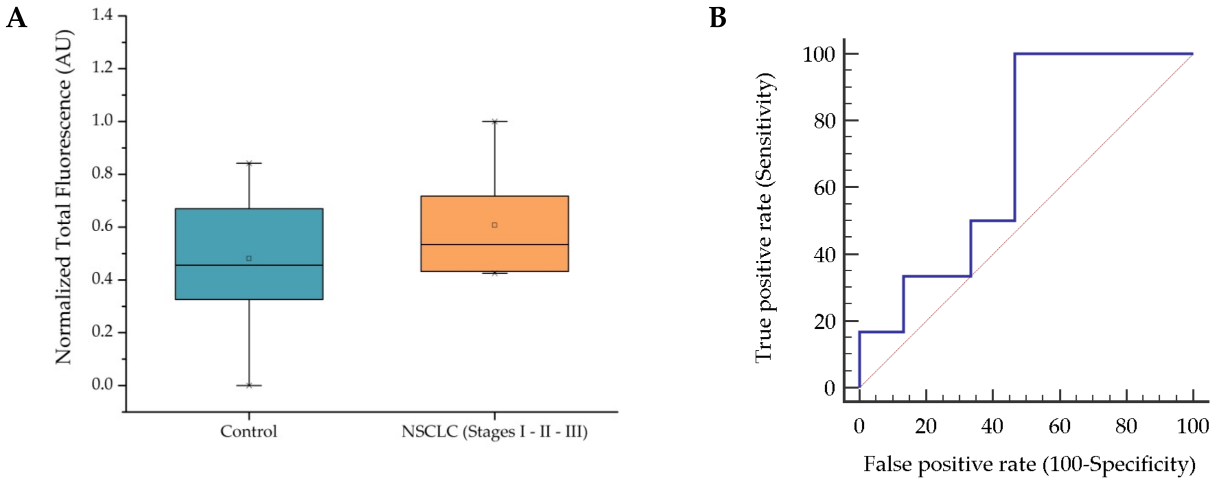

Figure S6. A. MB 21-5p hybridization studies with healthy and NSCLC PBMCs in stages I, II, and III. (mean ± SD). Data corrected for basal fluorescence and normalized to [0, 1]. Samples in study comprised: healthy control group (n=15); NSCLC stages I, II and III group (n=6), with NSCLC stage I (n=1), NSCLC stage II (n=2), and NSCLC stage III (n=3). **B.** ROC curve analysis on the developed MB 21-5p hybridization approach for miR-21-5p detection for healthy control and NSCLC PBMCs in stages I, II, and III.

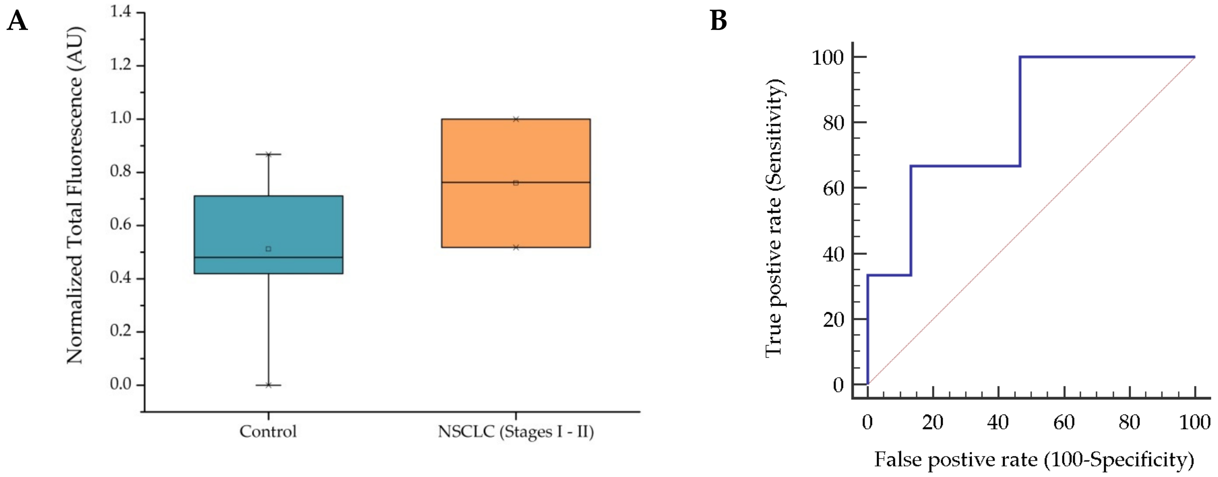

Figure S7. A. MB 21-5p hybridization studies with healthy and NSCLC PBMCs in stages I and II. (mean ± SD). Data corrected for basal fluorescence and normalized to [0, 1]. Samples in study comprised: healthy control group (n=15); NSCLC stages I and II group (n=3), with NSCLC stage I (n=1) and NSCLC stage II (n=2). **B.** ROC curve analysis on the developed MB 21-5p hybridization approach for miR-21-5p detection for healthy control and NSCLC PBMCs in stages I and II.
